# Brief telephone intervention for caregivers of medically complex patients during COVID-19

**DOI:** 10.1093/geroni/igaa057.3425

**Published:** 2020-12-16

**Authors:** Mary Stevens-Carr, Kevin Sethi, Channing Cochran, Margaret Bencomo-Rivera, Janice Marceaux

**Affiliations:** 1 South Texas Veteran Healthcare System, San Antonio, Texas, United States; 2 South Texas Veterans Health Care System, San Antonio, Texas, United States; 3 STVHCS, San Antonio, Texas, United States

## Abstract

The VA Home Based Primary Care (HBPC) program consists of an interdisciplinary team, including psychology, serving veterans with complex medical conditions who are supported by live-in caregiver(s). HBPC psychologists may work with caregivers to address caregiver stress. Some veterans enrolled in HBPC attend Adult Day Care (ADC) programs, allowing respite for caregivers. At the onset of COVID19 pandemic, ADC centers closed to minimize spread of the virus. The authors identified these caregivers to be at high risk for burnout and sought to develop a protocol to assist these caregivers via telephone and evaluate outcomes. PreCOVID-19 caregiver stress was known via a 4-item Zarit Caregiver Burden annual screening (Bédard et al., 2001). Following ADC closures, caregivers of veterans enrolled in ADC programs were contacted and re-administered the Zarit to determine impact of COVID-19 on caregiver stress. Caregivers of veterans not attending ADC were also contacted for comparison. Contacted caregivers were provided a brief CBT-based intervention via telephone, and post-intervention Zarit screening was administered after two weeks. Ultimately, 4 ADC caregivers and 4 non-ADC caregivers were contacted and provided with services before ADC centers reopened. Statistical analysis via mixed model ANOVA did not yield significant results, likely due to small sample size, although there was a large effect size (η_p^2 =.566). ADC caregivers generally reported increased stress from baseline following ADC closure and reduced stress following provision of intervention. The authors will present caregiver feedback about aspects of telephone intervention that were helpful, and not helpful, as well as authors impression.

